# “Role of radiology in a multidisciplinary approach to patient care”: summary of the ESR International Forum 2022

**DOI:** 10.1186/s13244-023-01377-x

**Published:** 2023-02-03

**Authors:** European Society of Radiology (ESR)

**Affiliations:** European Society of Radiology (ESR), Am Gestade 1, 1010 Vienna, Austria

**Keywords:** Multidisciplinarity, Teams, Visibility, Cooperation, Challenges

## Abstract

At the ESR International Forum 2022, held at ECR 2022 (July 16 in Vienna, Austria) different views, initiatives, and ideas from participating societies were presented with regard to the position and role of radiology in the changing landscape of health care and its role as an integral part of multidisciplinary teams. While it is unanimously agreed that multidisciplinarity is a key aspect of modern patient care, it creates new challenges that need to be addressed.

## Key points

- Multidisciplinary approach to patients is vital for providing balanced and comprehensive health care.
- Radiology is an integral and indispensable part of all multidisciplinary teams in diagnosing and treating patients.
- Radiologists need to collaborate with colleagues from other disciplines and increase their visibility.
- Despite numerous benefits, multidisciplinarity also creates new challenges for radiologists that need to be considered.
- Multidisciplinarity and building bridges between professions is the way forward as only this approach can ensure that patients will receive the best possible care.

## Introduction

The ESR International Forum was established by the European Society of Radiology (ESR) with the aim of discussing pivotal themes in the field of radiology with international societies from outside Europe. Therefore, non-European societies, with which the ESR has a Memorandum of Understanding with, and the International Society of Radiology as a global stakeholder are invited.

The ESR International Forum is held every year at the European Congress of Radiology (ECR) and participation is by invitation only. Previous topics discussed in the ESR International Forum include the relationship of radiologist and AI, measures undertaken by radiologist in the fight against Covid-19, the relation between radiology and nuclear medicine, the position of ultrasound in radiology, the relation of general radiology and subspecialty radiology, the implementation of clinical decision support and imaging referral guidelines in the clinical routine, the position of interventional radiology within radiology, value-based radiology, and the strategies to engage the younger generation.

For the meeting in 2022 the topic of multidisciplinary patient care was chosen by the ESR Executive Council in-line with the congress theme of ECR 2022 (“Building Bridges”) and in reflection of current developments in radiology and health care.

## Summaries of presentations

The following summaries represent the participating societies’ submitted written reports and presentations delivered during the meeting:

### The situation in North America

B. Haffty presented on behalf of the Radiological Society of North America (RSNA). Pointing out that the RSNA, in accordance with its mission statement, promotes excellence in patient care and health care delivery through education, research and technologic innovation, B. Haffty stated that RSNA currently has more than 47,000 professionals as members, including medical physicists, radiation oncologists, as well as radiologists. He further stated that radiology is at the core of patient preventative care, diagnosis, management, therapy, and follow-up and that the RSNA provides members with global collaboration, research support, networking, education, and workflow tools, striving to interact efficiently with referring clinicians and patients. The importance of multidisciplinarity is further reflected in the RSNA 2022 annual meeting theme: Empowering Patients and Partners in Care. Furthermore, B. Haffty presented the RSNA’s *Radiology Cares* initiative that connects radiologists with other health care professionals and patients, with imaging being just one component of the total patient experience. He explained that the essence of the RSNA’s mission are Research, Education, and Publication and presented initiatives such as the RSNA Research and Education Foundation, the Quantitative Imaging Biomarker Alliance (QIBA) as well as the family of authoritative RSNA journals, including *Radiology* and *RadioGraphic*. Presenting the Medical Imaging Data and Resource Center (MIDRC), B. Haffty emphasized that the RSNA has helped to accelerate the transfer of COVID-19 data and knowledge to aid physicians of all specialties in understanding the pathophysiology of this novel disease. Additionally, the RSNA Imaging AI Certificate programme, launched in 2022, delivers a pathway for radiologists to leverage artificial intelligence applications to expedite workflows and enhance patient care. Finally, B. Haffty presented the upcoming RSNA 2022 annual meeting where his Presidential Address will deal with “Diagnostic Imaging: Value from the Lens of the Patient.”

H.B. Fleishon, on behalf of the American College of Radiology (ACR), emphasized the ACR Appropriateness Criteria that assist referring physicians and other providers in making the most appropriate imaging decisions for specific clinical conditions. By employing these guidelines, providers and radiologists enhance safety and quality of care by contributing to the most efficacious use of radiology. The guidelines are developed and reviewed annually by expert panels in diagnostic imaging, interventional radiology with involvement of other specialists and primary care physicians. He further presented the ACR Practice Parameters and Technical Standards that provide radiologists, interventionalists, and radiation oncologists with consensus-based recommendations to help advance the science of radiology and reduce variability in care. Each practice parameter and technical standard is developed by subject matter radiology experts but also regularly include a multidisciplinary process. The ACR Reporting and Data Systems (RADS) provide standardized imaging findings terminology, report organization, assessment structure and classification for reporting, and data collection in patient imaging. These are developed in collaboration with non-radiologists, both from the US and international. As the leading advocacy organization for the USA radiology, the ACR organized a Coalition made up of over 120 medical professional organizations. This multi-specialty approach, which also includes non-physician providers, has widened the breadth and credibility of the ACR’s efforts to advocate on behalf of its members and its patients. H.B. Fleishon further presented ACR’s initiatives pertaining to population health as well as health equity. Finally, H.B. Fleishon stated that the Artificial Intelligence (AI) will also require a multidisciplinary approach to ensure not only its success but also patient safety. As a leader in AI, ACR has been working with multiple organizations not only within radiology but other specialties.

G. Soulez, on behalf of the Canadian Association of Radiologists (CAR), presented the most relevant trends influencing the future of radiology, including population aging, IT development, personalized and precision medicine, and free access to research results and data sharing. In order to overcome these challenges, the radiologists need to cooperate with colleagues from other disciplines. G. Soulez further presented the specificities of the Canadian environment, including a significant backlog for medical imaging procedures and limited access to medical imaging equipment. Commenting on the challenges and opportunities, G. Soulez pointed the need for a close collaboration with referring physicians and other specialties requiring frequent imaging or access to imaging to ensure that patients are receiving the best care. Internally, the CAR created four affiliate societies to help connect specialty radiologists, creating a place for them to collaborate on guidelines, education, and screening programs. The CAR is also working to make the referral process more efficient, ensuring the patient is getting the right test at the right time and is in the process of creating Canadian evidence-based referral guidelines with the goal of these being available to all referring health practitioners electronically. The CAR also runs several programs for quality assessment. G. Soulez emphasized that the CAR has established partnership with other radiology societies as well and national medical societies to advocate for shared priorities. Finally, G. Soulez presented the new CAR approach that is more inclusive and collaborative stating that the key priorities for the next 5 years will include guidelines, clinical implementation and validation of AI, education, community, and advocacy.

### The situation in Latin America

B.E. González Ulloa, representing the Interamerican College of Radiology (CIR), introduced the main roles and responsibilities of the CIR. She explained that one of the greatest issues for radiologists in a multidisciplinary workplace is that imaging touches practically every element of patient care. As imaging technology has evolved, the field of radiology has become more sophisticated and the radiologist must be familiar with the clinical situation in which the studies are being ordered, as well as the implications of the scan results for the patient. This degree of expertise is achieved through ongoing medical education, which has varied requirements for radiologists in each CIR member country. B.E. González Ulloa further pointed out that, as many locations have had to change their practice patterns due to the breadth and complexity of radiology and nuclear medicine, radiologists have had to specialize in order to perform effectively in a multidisciplinary context. The CIR has adopted a multidisciplinary disease-based strategy in response to this movement in clinical practice toward interdisciplinary care. However, the CIR must also keep in mind that the vast majority of radiologists in its member countries are generalists rather than subspecialists and the substance and depth of information must be adapted to unique needs. To tackle these challenges, the CIR has built numerous venues to provide current and relevant continuous medical education, including a website, webinars, workshops, courses, and specific issue gatherings. Lastly, B.E. González Ulloa stated that another significant achievement of the CIR over the years has been the collaboration between radiologists from all member countries in the organization of meetings, seminars, case-based teaching, web pages and workshops to discuss the various future scenarios that CIR countries will face, such as technology, artificial intelligence, and big data.

G.M. Figueroa Sanchez, on behalf of the Mexican Federation of Radiology and Imaging (FMRI), reported on the current situation in Mexico. He pointed out that the FMRI is committed to a multidisciplinary approach, which is one of its core practices. FMRI impacts both academic communication among colleagues and the improvement in patient care and has, for many years, promoted numerous activities with a multidisciplinary approach, such as multidisciplinary participation in national meetings and congresses, promotion of a multidisciplinary approach in radiology education and residency curricula, inclusion of public, private, and academic specialties and most recently, the launching of the Journal of the Mexican Federation of Radiology and Imaging (JMeXFRI). Furthermore, G.M. Figueroa Sanchez explained that, at each meeting or webinar that FMRI organizes, there are presentations and roundtable discussions involving clinicians (e.g., surgeons, neurologists, and oncologists) and the FMRI radiologists take part in meetings of other specialties. G.M. Figueroa Sanchez also pointed out the value of educating the public and patients about the importance of a multidisciplinary approach for the correct interpretation of imaging studies and the application of their results in the clinical context of each patient, primarily through the FMRI website, but also in TV, radio broadcasts, and social media. Each time the FMRI has the opportunity to work with professors of radiology residency programs, it emphasizes the importance of communication between clinicians and radiologists and the FMRI has for many years now promoted the idea of the "visible radiologist" with all its implications and responsibilities.

N. Rodríguez Pedraza on behalf of the Mexican Society of Radiology and Imaging (SMRI), stated that the requests for radiological studies to support a diagnosis have increased considerably. At the same time, old diagnostic methods in radiology will not be functional in the future, as no single medical specialty will be able to provide patients with all their health care needs without a multidisciplinary approach. She emphasized the importance of the participation of radiologists in Multidisciplinary Team (MDT) meetings. Talking about the current situation in Mexico, N. Rodríguez Pedraza explained that these meetings are done in different ways in most private hospitals and third level hospitals. Some hospitals bring together radiologists, surgeons, clinicians, and pathologists to discuss an important case, other hospitals have meetings based on a specialty area, while her hospital organizes daily meetings with residents, to analyze cases that requires more attention. Pointing out the advantages and disadvantages of such meetings, she stated that MDT meetings improve the quality of patient care and management and serve to generate a learning platform, whereas the use of PACS’ equipment facilitates the review of images and helps to reduce the time in data retrieval and cross-referencing information for diagnosis. On the other hand, MDT meetings can be time consuming, especially in environments with a higher workload. In conclusion, N. Rodríguez Pedraza stated that radiology departments will continue to play a pivotal role in MDTs.

L.A. Cruz, reporting on behalf of the Colombian Association of Radiology (ACR), stated that radiologists should not be considered just another link in the chain of patient care but have a leading role with proactive participation. To achieve this, he pointed out, it is important to change the mental perception of the radiologist and to encourage more young radiologists to enter into interventional radiology, since it is an area of the specialty that allows a broader and more complete approach to patient care, covering both diagnosis and treatment. This can be achieved through an adequate training of the radiologist, starting from residency, complemented by permanent continuing medical education activities, which ideally should be provided by the national scientific societies of the specialty. Finally, L.A. Cruz stated that multidisciplinary approach to patient care is part of professionalism, which is the set of fundamental beliefs and values that guide the daily work of physicians and includes different ethical responsibilities, such as suitability, informed consent, patient protection, communication with patients and physicians, continuous learning, continuous quality improvement and minimizing unnecessary studies. The professionalism of the radiologist is one of the pillars promoted by the Colombian Association of Radiology among their members.

V. Muglia, on behalf of the Brazilian College of Radiology (CBR), presented the situation in Brazil. He explained that with the massive development of imaging technology, radiologists started joining multidisciplinary teams in tertiary health care centers. This approach has become a cornerstone of what is referred to as Value-Based Health care. V. Muglia pointed out, however, the “paradox of technology” that might lead to decreased interactions with patients and other professionals, which is something multidisciplinary rounds contribute to alleviating. To overcome this paradox, a variety of consultation services and multidisciplinary team interactions have been described and adopted. There are different ways for organizing second opinion services, hybrid consultations, periodic multidisciplinary conferences and tumor boards. Also, some applications were designed to enhance interdisciplinary consultations. V. Muglia further mentioned that the maintenance of a central role will rely on collaboration and effectiveness and that, besides technical skills, radiologists should be comfortable with other aspects of health care, including but not limited to cost-effectiveness of radiological interventions, patient’s safety and quality control metrics. Lastly, V. Muglia presented the activities done in this regard by the CBR, including guides and simulations for members on participating in tumor boards and multidisciplinary teams in general.

C. Higa Nomura submitted a report on behalf of the Radiological and Diagnostic Imaging Society of Sao Paulo (SPR). He pointed out the importance of change from volume-based care to value-based care. To achieve this, radiologists will have to play a more significant role in medical decision support, mainly through participation in multidisciplinary teams. As such, the modern radiologist is a gatekeeper who discusses diagnoses and treatments with other professionals and, sometimes, with the patients themselves. Some areas, such as interventional radiology, by their nature, interact more directly with patients and the clinical staff. C. Higa Nomura reported that in Brazil, all ultrasound exams are performed by radiologists who participate directly in the clinical decision-making process. Radiologists are also featured in tumor boards where they play a fundamental role in conducting the diagnosis and treatment of the patient. Additionally, radiologists have become indispensable in heart teams where they define the best treatment for a patient with valve disease. C. Higa Nomura provided examples of Brazilian radiologists working with heart teams to determine whether the patient should undergo surgery or transcatheter aortic valve implantation (TAVI).

### The situation in Egypt

T. El-Diasty, on behalf of the Egyptian Society of Radiology and Nuclear Medicine (ESRNM), stated that it is predicted that the multidisciplinary approach would substantially increase in the future through the use of MDT meetings. El-Diasty, however, pointed out that there are several challenges related to these meetings. The preparation, attendance and follow-up time required for MDT meetings is considerable, especially when supervising radiology trainees involved in the meeting. Preparation should be accounted for in a radiologist’s workload. Late additions to lists of patients for discussion at MDT meetings may compromise accuracy of image interpretation and prevent adequate preparation by clinical radiologists. MDT meetings necessitate a great deal of organization and infrastructure to ensure the presence of relevant personnel, the collection and compilation of important patient details and radiological data. There can exist a lack of easy access to imaging studies performed outside of the institution at which the MDT meeting is conducted. Opportunity costs to the hospital or practice resulting from clinical radiologists being taken away from more highly remunerated activities should also be considered. Presenting the main ESRNM recommendations, T. El-Diasty emphasized that participation in MDT meetings is an important component of training to become a clinical radiologist and is strongly supported by the organization. He also pointed out the importance of proper documentation and following deadlines for submission of patient details and access to imaging studies prior to the meeting to allow the radiologist adequate preparation time. In conclusion, T. El-Diasty stated that efforts should be made to reduce the effects of workload and stress on the radiologist by taking measures such as incorporating these meetings during the work hours, by increasing the strength of department and by proper coordination between physicians and radiologists.

### The situation in Saudi Arabia

A. Badeeb, on behalf of the Radiological Society of Saudi Arabia (RSSA), explained that MDT meetings have become an integral part of patient care in Saudi Arabia, with radiologist being an indispensable part of those teams. Additionally, radiologists play an important role in MDT research meetings as well. Examples include MDTs concerning gastrointestinal tumors, inflammatory bowel diseases, pulmonary diseases, breast cancers, sarcomas, gynecologic oncology, lymphoma, ENT and many more. The RSSA has taken part in numerous multidisciplinary workshops, organized not only by the RSSA but also by the society of interventional radiology as well as the industry. The RSSA also undertakes various activities to increase the visibility of radiologists not only within the hospitals but also among the general public.

### The situation in the Asia-Oceania region

E. Ho, on behalf of the Asian Oceanian Society of Radiology (AOSR), stated that radiologists are integral and indispensable for optimal health care delivery and that the AOSR’s goal is the right care delivered to the right patient at the right time in the right place. E. Ho further presented main AOSR recommendations for radiologists, including taking part in clinical multidisciplinary teams and engagement with other clinicians even in “kerbside” discussions where no formal team has been set up, active involvement in the development and application of Clinical Practice Guidelines, Consensus Statements and Clinical Decision Support. Other recommendations include joining hospital wide Safety and Quality Activities, Mortality and Morbidity conferences and others, as well as advocating recognition of radiologists’ roles in multidisciplinary teams and activities, including that in the job scope (even setting up KPIs), where possible. E. Ho explained that this multidisciplinary approach can help identify health care gaps, optimize use of imaging technology and intervention to arrive at an accurate diagnosis and/or intervention and coordinate patient care, faster time to patient care and provide a better outcome. When establishing multidisciplinary teams, it is important to identify committed leaders, clear goals and disciplined time keeping, foster trust and respect, effective communication and collaboration and audit effectiveness. Well-setup teams can serve as continuing education platforms. E. Ho also identified main challenges to this approach including time, manpower, varied subspecialties and IT and administrative support. Finally, E. Ho presented main AOSR multidisciplinary activities including AOSR Structured Template Reporting (ASTeR), Value-Based Radiology (VBR) and Artificial Intelligence Applications in Radiology.

### The situation in Thailand

W. Tanomkiat, submitted a report on behalf of the Royal College of Radiologists of Thailand (RCRT). He stated that the RCRT in close collaboration with the Radiological Society of Thailand (RST) and related societies emphasizes multidisciplinary radiology team working to its members in two ways: within the curriculum for residents (who are mandatory associate members) and during the annual congress for all members. In the curriculum, the RCRT in collaboration with the Thai Medical Physicists Society introduces radiation biology and physics and the role of physicists in radiation safety within the first year of training. The residents are also trained to develop non-technical skills and multidisciplinary integration that are essential in working in a team with other professions at their training institutes after attending instruction courses held by the RCRT. In the RCRT-RST annual congress, parallel multidisciplinary sessions are regularly practiced in all three days of the congress. RCRT also has an important role in a multidisciplinary approach to patient care especially in major health problems of Thailand, such as during the COVID-19 pandemic [1, 2]. The RCRT guides how to estimate disease and fibrotic extents which is part of the important information in deciding to start anti-fibrotic therapy in idiopathic pulmonary fibrosis and systemic sclerosis [3–5]. RCRT works closely with the Thoracic Society of Thailand under the Royal Patronage and the Foundation of Orphan and Rare Lung Disease (FORLD) in establishing a national HRCT protocol, checklists, and guidelines in caring, diagnosis, and treatment of interstitial lung disease.

J. Euathrongchit, on behalf of the Radiological Society of Thailand (RST), reported that modern medicine involves advances in technology, including advanced computational methods integrated into imaging services. Key trend of modern medicine is image centricity with a focus on precision and a personalized diagnosis and treatment concept, with radiology being an integral part of this approach. Through its activities, the RST has become a backbone of the Thailand’s multidisciplinary approach to patient care. J. Euathrongchit further presented the main activities in this regards, including reaching out to professional groups in radiological services, such as radiographers and medical physicists to strengthen collaboration among the professions, active use of social media and electronic platforms to provide continuing education activities for radiologists, teaming up with the sister-organization, the Royal College of Radiologists of Thailand (RCRT) to strengthen education, research, and professional practice activities, and reaching out to international counterparts, such as regional and international radiological societies to establish the networking for mutual collaboration in all aspects of the radiological profession.

### The situation in Japan

K. Yamada, on behalf of the Japan Radiological Society (JRS), reported that the medical system in Japan is characterized by low costs (being under government control and run under planned economy) and free access (patients allowed to choose any institution in the country). However, certain weaknesses of this system have been identified, including a lack of standardization and specialization—nuclear medicine is not separated from radiology, radiation oncology is not separated from radiology, and non-radiologist physicians also read cases. Further weaknesses identified and presented by K. Yamada include that the number of specialists is not controlled due to a strong influence of the Japan Medical Association (JMA) that is general practitioner (GP) driven and GPs in Japan, on average, earn a lot more than specialists. The reason for this disbalance in payment is because GPs are paid on a fee-for-service basis while specialists have a fixed salary. Furthermore, K. Yamada explained that this situation has led to an increase in members in individual departments, in accordance with the principles of behavioural economics. Such an environment will favor those with a strong voice, and in the case of Japan, this has led to a very low number of radiologists per capita, which is an issue that needs to be addressed. K. Yamada stated that, due to the lack of control in number of specialists in Japan, radiologists are outnumbered by surgeons, and this leads to a self-referral practice. The role of radiologists in this multidisciplinary approach, as explained by K. Yamada, will have to include gatekeeping through careful observations of what transpires in the hospitals and attending conferences, strong focus on quality control issues, reducing number of unnecessary examinations, and consequently reducing patients’ exposure to radiation. K. Yamada also pointed out that radiologists need to focus on additional standardization and specialization.

### The situation in Korea

J. M. Lee, on behalf of the Korean Society of Radiology (KSR), presented the current situation in Korea. He provided an overview of the multidisciplinary care (MDC), stating that in the current medical insurance system in Korea, MDC for cancer patients is mainly covered, although this may expand to other diseases in the future; the cost of MDC for cancer patients is determined by the number of participating specialists. MDC is usually done in an outpatient setting but can be of great help to inpatients as well. The percentage of MDC is included as an important indicator in the Cancer Adequacy Evaluation conducted by the Health Insurance Review and Assessment Service. As a result, many hospitals try to provide MDC for cancer patients, although it is limited due to a shortage of manpower. In addition, the role of a radiologist in MDC is sometimes limited, because the actual conversations with the patients and decision-making process is carried out by the attending physician or one senior physician. J. M. Lee pointed out that, in order for radiologists to successfully participate in MDC, evaluation of and adequate compensation for the additional workload caused by MDC are necessary. Although rewarding, participating in MDC takes extra time and effort for radiologists who already have heavy workloads. In order to increase treatment efficiency, subdividing MDC teams according to the purpose of treatment may be helpful. He concluded that the multidisciplinary approach is an important form of practice pattern for radiologists, in which so-called invisible radiologists play an important role as visible doctors who directly participate in patient treatment. It is also an opportunity for radiologists to be face-to-face with patients and take the lead in the clinical decision-making process. In many hospitals, it may be difficult for radiologists to participate in MDC due to heavy workloads; however, those who can should participate, bearing in mind that the presence of radiologists is paramount in maintaining the quality of MDC.

### The situation in Europe

R. Beets-Tan reported on behalf of the European Society of Radiology (ESR). She presented the view of ESR members on the topic of multidisciplinarity and their role, expressed in the recently published article *The role of radiologist in the changing world of health care—a White Paper of the ESR* [6] as well as the earlier paper published in 2020 in Insights into Imaging—*The identity and role of the radiologist in 2020: a survey among ESR full radiologist members* [7]. R. Beets-Tan further presented the ESR’s recommendations and actions, including continuous support for clinical and sub-specialty training, a communication strategy, guidance for integration of multidisciplinary team working, and enhancing the visibility of radiology by highlighting radiology-led research and education, including undergraduate training in radiology. A significant step toward understanding the importance of multidisciplinarity is also the ECR 2022 congress with its theme of building bridges between radiology and other medical disciplines, patients, scientists, radiographers, industry partners, between ESR and other medical societies to improve patient care. With such a strong focus on multidisciplinarity, the congress featured over 300 educational sessions, 100 multidisciplinary and interactive with more than 100 speakers from outside radiology including leaders from medical societies, EU policy makers, tech, and pharma industry.

### Position of the International Society of Radiology (ISR)

R.A. Mendonça, on behalf of the International Society of Radiology (ISR), reported that the ISR promotes the multidisciplinary approach through a strong collaboration with different stakeholders, the IAEA and the WHO. He pointed out that the ISR Quality and Safety Alliance (ISRQSA), which aims to facilitate an international quality and safety agenda, is composed of quality and safety campaigns from across the globe. These are mostly radiology-led multi-stakeholder organizations that include also medical physicists and radiographers/radiological technologists. By acting as a convener of and facilitator for these campaigns, the ISR is able to promote radiological quality and patient safety not only with the WHO and the IAEA, but also other stakeholders such as the International Council for Radiation Protection (ICRP), the International Organization for Medical Physics (IOMP), the International Radiation Protection Association (IRPA), the International Society of Radiographers and Radiological Technologists (ISRRT), the World Federation of Pediatric Imaging (WFPI), and the World Federation for Ultrasound in Medicine and Biology (WFUMB). Additionally, R.A. Mendonça stated that the ISR elevated its long-standing collaboration with the IAEA in 2021 by signing Practical Arrangements on cooperation in diagnostic and interventional radiology and that the ISR will participate as a cooperating organization in the IAEA International Conference on Integrated Medical Imaging in Cardiovascular Diseases (IMIC-2022) in December 2022, Vienna/AT, an event targeted to NM physicians, radiologists, cardiologists, radiochemists/radiopharmacists, medical physicists, technologists/radiographers, and other cardiac imaging experts. Multidisciplinary efforts also take place through the WHO, with whom the ISR renewed its Work Plan for 2022–2024. After having collaborated in the WHO Rapid Advice Guide on the Use of chest imaging in COVID-19, which involved experts and stakeholders from manifold disciplines such as guideline methodology, emergency medicine, intensive care, pulmonology and molecular diagnostics, and patient advocacy in addition to medical imaging, the ISR and the WHO organized further joint webinars. In conclusion, R.A. Mendonça stated that multidisciplinarity is one of the core principles the ISR pursues in working toward improving patient care. Through its collaboration with the WHO, the IAEA and other high-ranking international organizations, the ISR continuously raises awareness of the vital role medical imaging and interdisciplinary collaboration play in improving global health.

## Discussion and conclusion

Multidisciplinary care teams are composed of members from different medical specialties working together to ensure the highest quality of care for the patient. With the evolution of health care, multidisciplinary approach has become indispensable, and have moved radiologists to a central role in contributing to clinical decision-making.

For best patient care, radiologists must engage in direct and frequent interaction with other members of the care team to be aware of the clinical scenario, imaging referrals and follow-up, as well as results and implications for the patient. The increasing power of the different imaging methods and the development of Interventional Radiology have expanded the direct involvement of radiologists in patient management [8].

Due to a growing complexity in diagnostic imaging requiring radiologists to take more responsibility and commitment in the MDT, the radiologists play a major role in the provision of care and in the MDT’s negotiation. With thorough preparation active communication and discussion, providing detailed reports and professional expertise, the radiologists become able to organize and guide a consensus-based decision from that new central position [9].

This increased demand for radiologists to take part in multidisciplinary care is particularly challenging in light of the ever-increasing workload and consequently limited resources.

## Data Availability

All data generated or analyzed during this study are included in this published article.

## Abbreviations

ACR: American College of Radiology
ACR: Colombian Association of Radiology
AI: Artificial intelligence
AOSR: Asian Oceanian Society of Radiology
ASTeR: AOSR Structured Template Reporting
CBR: Brazilian College of Radiology
CIR: Inter-American College of Radiology
ECR: European Congress of Radiology
ESR: European Society of Radiology
ESRNM: Egyptian Society of Radiology and Nuclear Medicine
FMRI: Mexican Federation of Radiology and Imaging
FORLD: Foundation of Orphan and Rare Lung Disease
GP: General practitioner
IAEA: International Atomic Energy Agency
ICRP: International Council for Radiation Protection
IMIC-2022: IAEA International Conference on Integrated Medical Imaging in Cardiovascular Diseases
IOMP: International Organization for Medical Physics
IRPA: International Radiation Protection Association
ISR: International Society of Radiology
ISRQSA: ISR Quality and Safety Alliance
ISRRT: International Society of Radiographers and Radiological Technologists
JMA: Japan Medical Association
JMeXFRI: Journal of the Mexican Federation of Radiology and Imaging
JRS: Japan Radiological Society
KPI: Key Performance Indicator
KSR: Korean Society of Radiology
MDC: Multidisciplinary care
MDT: Multidisciplinary team
MIDRC: Medical Imaging Data and Resource Center
MOU: Memorandum of Understanding
RADS: Reporting and Data Systems
RCRT: Royal College of Radiologists of Thailand
RSNA: Radiological Society of North America
RSSA: Radiological Society of Saudi Arabia
RST: Radiological Society of Thailand
SMRI: Mexican Society of Radiology and Imaging
SPR: Radiological and Diagnostic Imaging Society of São Paulo
TAVI: Transcatheter aortic valve implantation
VBR: Value-based radiology
WFPI: World Federation of Pediatric Imaging
WFUMB: World Federation for Ultrasound in Medicine and Biology
WHO: World Health Organization
